# Bone and Mineral Disorder in Renal Transplant Patients: Overview of Pathology, Clinical, and Therapeutic Aspects

**DOI:** 10.3389/fmed.2022.821884

**Published:** 2022-03-10

**Authors:** Paolo Molinari, Carlo Maria Alfieri, Deborah Mattinzoli, Mariarosaria Campise, Angela Cervesato, Silvia Malvica, Evaldo Favi, Piergiorgio Messa, Giuseppe Castellano

**Affiliations:** ^1^Department of Nephrology, Dialysis and Renal Transplantation, Fondazione IRCCS Ca' Granda Ospedale Policlinico, Milan, Italy; ^2^Department of Clinical Sciences and Community Health, University of Milan, Milan, Italy; ^3^Renal Research Laboratory Fondazione IRCCS Ca' Granda Ospedale Policlinico, Milan, Italy; ^4^Department of Nephrology, Clinical and Translational Sciences, Università degli Studi della Campania L.Vanvitelli, Naples, Italy; ^5^Department of General Surgery, Renal Transplantation, Fondazione IRCCS Ca' Granda Ospedale Maggiore Policlinico, Milan, Italy

**Keywords:** mineral disorders, bone disorders, renal transplantation, graft outcome, CKD-MBD treatment

## Abstract

Renal transplantation (RTx) allows us to obtain the resolution of the uremic status but is not frequently able to solve all the metabolic complications present during end-stage renal disease. Mineral and bone disorders (MBDs) are frequent since the early stages of chronic kidney disease (CKD) and strongly influence the morbidity and mortality of patients with CKD. Some mineral metabolism (MM) alterations can persist in patients with RTx (RTx-p), as well as in the presence of complete renal function recovery. In those patients, anomalies of calcium, phosphorus, parathormone, fibroblast growth factor 23, and vitamin D such as bone and vessels are frequent and related to both pre-RTx and post-RTx specific factors. Many treatments are present for the management of post-RTx MBD. Despite that, the guidelines that can give clear directives in MBD treatment of RTx-p are still missed. For the future, to obtain an ever-greater individualisation of therapy, an increase of the evidence, the specificity of international guidelines, and more uniform management of these anomalies worldwide should be expected. In this review, the major factors related to post-renal transplant MBD (post-RTx-MBD), the main mineral metabolism biochemical anomalies, and the principal treatment for post-RTx MBD will be reported.

## Introduction

Renal transplantation (RTx) is the best therapy for chronic kidney disease (CKD). In this context, RTx permits us to obtain the resolution of the uremic status. Unfortunately, RTx is not able to solve all the metabolic complications present during end-stage renal disease (ESRD).

Mineral and bone disorders (MBD) are frequent from the early stages of CKD and strongly influence the morbidity and mortality of CKD patients. In the presence of an RTx, some mineral metabolism (MM) alterations can persist, also in the presence of complete renal function recovery. These anomalies mainly involve calcium (Ca), phosphorus (P), vitamin-D, and parathormone (PTH), and are responsible for post-renal transplant MBD (post-RTx-MBD). Similar to CKD, also in RTx patients (RTx-p), the MBD can lead to several bone changes in quality and density, thus, determining an important increase in cardiovascular and fracture risk.

In the present review the major factors related to post RTx-MBD, the main MM biochemical anomalies, and the principal treatment for post-RTx MBD will be described.

## Mineral and Bone Disorder in RTx

The impact of MBD in patients with RTx is a matter of serious concern. In fact, despite some data reporting a significant bone loss in only 0.1–5.7% of RTx-p, the fracture risk in RTx-p is 5 times higher than in the general population. This risk, present thoughout all the RTx life, is significantly higher during the first 5 years of RTx (1, 2).

The prevalence of MBD in RTx-p could be much higher than previously thought and, therefore, considering the medical complexity of these patients, this process has to be seen as a wide spectrum of risk factors and clinical, biochemical, and histopathologic alterations.

Some data have reported a correlation between fracture events and the risk of mortality. In 2020, Iseri et al. analysed data from the Swedish National Renal Registry of 3,992 first RTx recipients. According to their data, a crude incidence rate of the first episode of major fracture that is present in 279 RTx-p was 13.5/1,000 patient-years, with hip fractures reported in 69 RTx-p in 3.4/1,000 patient-years. The incidence rates were highest during the first 6 months following RTx, and the presence of the first episode of major hip or spine fracture independently predicted an increased all-cause mortality risk (hazard ratio, HR, 1.78) (3).

However, it is important to underline that post-RTx-MBD is not only certainly related to RTx status but is also certainly influenced by the patients in pre-RTx condition.

### Pre-transplant Factors Related to RTx-MBD

In Table 1, the main pre-RTx factors related to MBD insurgence during transplantation are listed. Among general factors, certainly, the principal modifiable factor is dialysis vintage. In a recent paper published by Sutton et al., several parameters are present in almost 20% of RTx-p, including dialysis vintage, older age, and the presence of severe pre-RTx hyperparathyroidism (HPT) and influence the development of post-RTx HPT. The persistence of this anomaly indicates a pre-RTx MBD of a severe degree (4). This fact is of high relevance and underscores the need to initiate the study for RTx in ESRD as soon as possible, possibly performing living and/or pre-emptive RTx.

**Table 1:** Pre-transplant factors associated with MBD development after RTx.

**General factors**
Female gender, especially post-menopause women
Age ≥ 50 years old
Diabetes
Dialysis vintage
**Osteoporosis**
Younger age at transplantation
Malnutrition
Smoking
Alcohol abuse
Drugs (heparin, warfarin)
Glucocorticoids before renal transplantation
**Fracture**
Pre-RTx osteoporosis
Pre-RTx renal osteodystrophy
Pre-RTx fractures

*RTx, renal transplantation*.

An association between bone disease in ESRD and poor nutritional status has been reported in relation to the increase of both intimal and medial vascular calcification and vascular senescence (5). This recent evidence not only increases the cardiovascular and mortality risk in pre-RTx time but also affects the early and long-term RTx outcome (6). This could also explain why some reports indicate that younger age at transplantation is associated with an increase in osteoporosis insurgence risk. This is due to an interplay between the precocious exposure to the uremic toxin and accelerated cellular senescence induced by uremia, and, after RTx, to the administration of glucocorticoid (GC). In fact, from an early age, GC exposure leads to a greater lifetime cumulative GC dose in comparison to patients who are transplanted at an older age. An increased risk of osteoporosis in patients with ESRD has also been related to the long-term use of unfractionated heparin (inhibition of osteoprotegerin and enhancing osteoclastic bone resorption) and warfarin (inhibition of gamma-carboxylation of osteocalcin). All these factors could determine a progressive decrease in bone density (7).

Pre-existing renal osteodystrophy negatively impacts the bone-related and general outcomes in RTx-p (8, 9). Several studies supported this hypothesis such as in the work of Iyer et al., where a cohort of 47 RTx-p glucocorticoids (GCs) was suspended in the first 3 days after RTx. Nevertheless, bone mineral density (BMD) significantly decreased at distal radius at 12 months, while lumbar and hip BMD were not affected (10). This may be due to the persistence of pre-existing renal osteodystrophy, with inappropriately high levels of PTH mainly affecting the compact cortical bone of long bones. In a recent study published by Lee et al., in a multivariate Cox analysis, the pre-RTx osteoporosis and osteopenia were independent risk factors for fracture in 941 RTx-p (HR 11.76 and 5.21, respectively) (11).

### Post-transplant Factors Related to RTx-MBD

**Figure 1:**
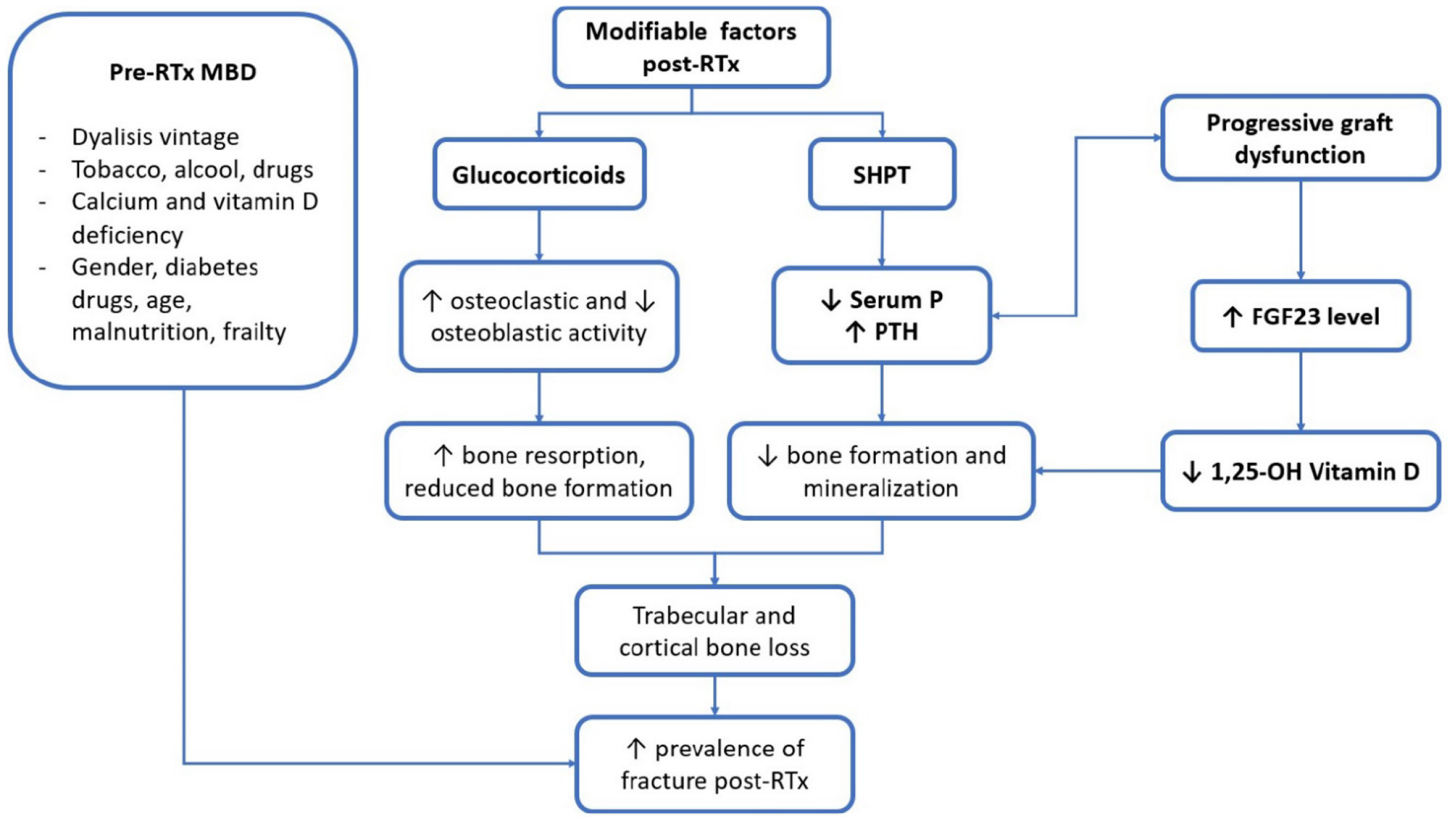
Main clinical and biochemical factors of patients with renal transplantation (RTx-p) mineral metabolism. RTx, renal transplantation; SHPT, secondary hyperparathyroidism; P, phosphorus; PTH, parathormone; FGF23, fibroblast growth factor 23; 1, 25-OH vitamin D, 1, 25 hydroxylated vitamin D.

In the following paragraphs, the main post-RTx factors related to RTx-MBD will be discussed (Figure 1). In particular, we will focus on the alterations in biochemical parameters, which is a crucial point in the evaluation of MBD in RTx-p, and then, we will discuss the problems related to bone disease and vascular calcification (VC). It is important to note that, before a therapeutic setting, all of these three points should be individually characterised.

#### Biochemical Parameters

**Figure 2:**
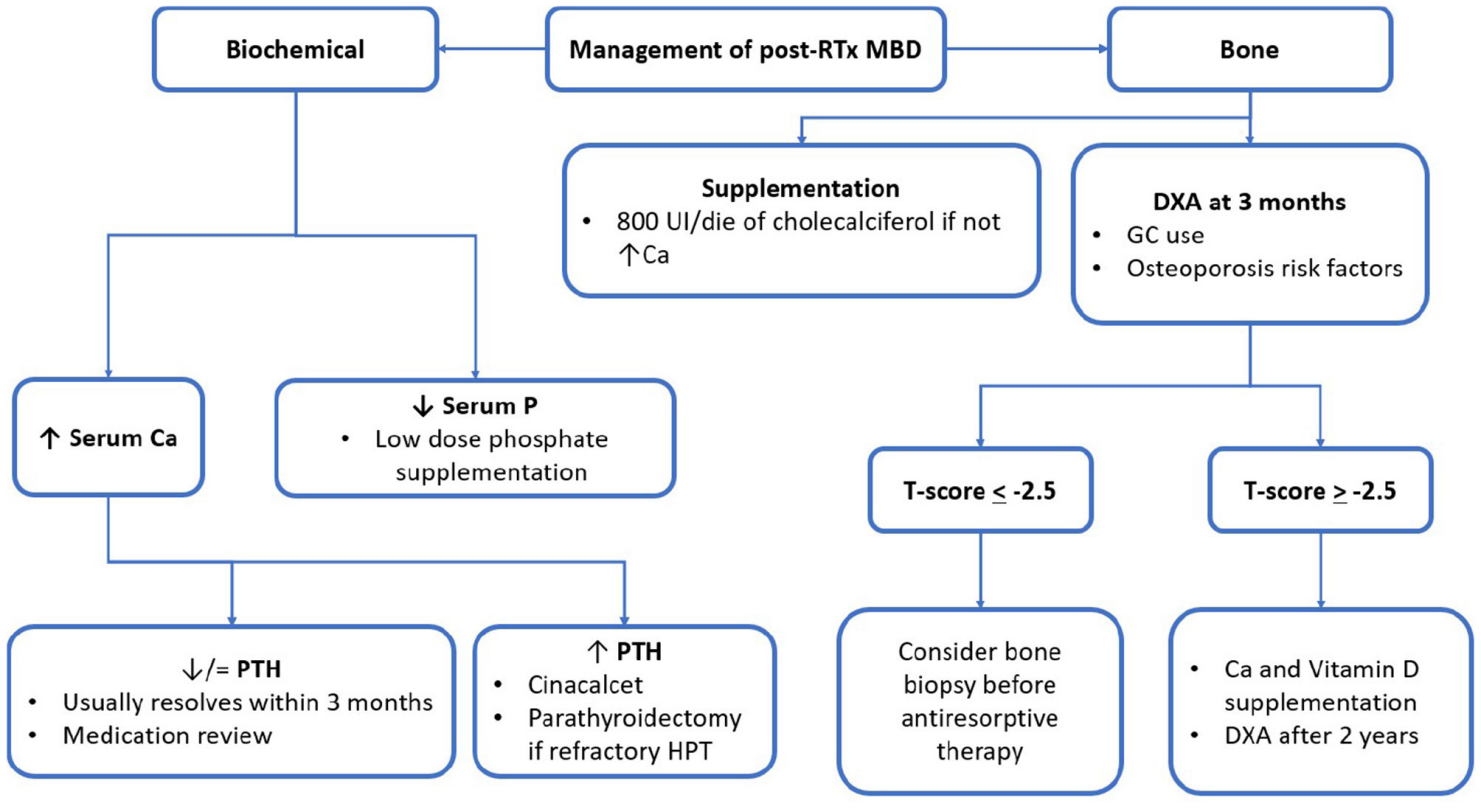
post-RTx MBD monitoring and management algorithm. RTx, renal transplantation; MBD, mineral bone disorder; GC, glucocorticoid; UI, international unit; eGFR, estimated glomerular filtration rate; Ca, calcium; P, phosphorus; PTH, parathormone; ABD, adynamic bone disease; DXA, dual-energy absorptiometry.

Biochemical parameters of MM are subject to important modifications after RTx. In Figure 2, the main factors and their interrelationship in influencing RTx-MBD development are represented. Despite their correlation with mortality for cardiovascular and all-causes events, it is still debated whether these factors may influence the outcome of graft (12). This subject needs to be better clarified by future interventional studies.

##### Calcium

Usually, serum calcium has a biphasic trend after RTx with an early initial decrease which is probably due to the significant fall in PTH seen after RTx. After that, calcium levels tend to rise, following the increased production of 1,25-OH vitamin D by a healthier kidney and the frequent persistence of HPT. The prevalence of hypercalcemia (hyper-Ca) is reported to be around 10–15% and will reach its maximum in 3 to 6 months after RTx (13). In our experience, a condition of hyper-Ca, defined for Ca levels > 10.4 mg/dl, is present in almost 25% of RTx-p during the first year of RTx. In most cases, hyper-Ca is related to high PTH levels. This condition can be associated with interstitial microcalcifications and potentially impact negatively on graft outcome (4). A work by Thomas et al. showed a negative impact of high calcium level on estimated glomerular filtration rate (eGFR) decrease, especially if hyper-Ca is associated with low P levels (14).

##### Phosphate

The serum phosphate levels markedly decrease in about 50% of patients after RTx. In our experience, *P* < 2.4 mg/dl is present in about 50% of RTx-p, after the first month of RTx. A similar condition is present in 15 and 12% of RTx-p after 6 and 12 months of RTx, respectively. Hypo-phosphatemia (Hypo-P) has several causes in RTx-p. First of all, it reflects an improvement in kidney function, which involves a better tubular sensitivity and responsiveness to PTH and fibroblast growth factor-23 (FGF-23) action, which is, sometimes, associated with inappropriately high PTH levels (7). In addition, it is important to remember the effect of some immunosuppressive drugs, such as calcineurin inhibitors (CNIs), that are associated with tubule toxicity and can be the principal reason for the development of Hypo-P in these patients (15).

Abnormalities in P metabolism have been associated with a decrease in osteoblast activity and defective bone mineralization. Post-RTx Hypo-P can have a detrimental effect on bone mineralization and might contribute to impaired osteoblast genesis and early osteoblast apoptosis, which lead to osteoporosis insurgence and progression. In this case, it seems that higher PTH levels exert protective effects on bone by preserving osteoblast survival (16).

##### PTH and FGF23

Within the first 12 months post-RTx, an initial decrease in the PTH levels is usually found (17). Of note, in up to 50% of cases, as well as in the presence of a well-functioning RTx, a persistent increase of PTH can be present (18). In our experience, the prevalence of high levels of PTH, considering the level of eGFR, in a cohort of 600 RTx-p at 24 months after RTx is around 48%. These data are almost in line with those reported in the literature (19). The impact of persistent HPT on graft outcome is still debated. In a study involving 1,609 RTx-p, persistent HPT was associated with the worse graft survival (10). Nevertheless, the optimal PTH value to achieve in RTx-p is still a matter of debate. The problem of secondary and tertiary HPT in RTx has been deeply presented in a recent paper published by our group (20).

The FGF-23 levels are high in RTx-p during the first 6 months after RTx and are significant in the development and maintenance of low P levels. At the moment, some recent evidence has reported a relationship in RTx-p of FGF-23 levels of RTx with anaemia status and cardiovascular risk (21, 22).

In any case, regarding the RTx-p, the FGF-23 utility in clinical practise is still debated.

##### Vitamin D

Native vitamin D (25-OH-VitD) is frequently below the sufficient levels in RTx-p. This fact reflects the pre-RTx status but is also related to some RTx specific factors. The evaluation of 25-OH-VitD and active vitamin D form (1-25-OH-VitD) is important in RTx-p. Increased availability of 1-25-OH-VitD might contribute to the development of hyper-Ca by the induction of renal 1-α-OH-ase activity (23). Of note, low levels of 25-OH-VitD in RTx-p are very frequent especially in the early phases of KTx (24). The persistence of low levels of 25-OH-VitD is determined by the need to reduce the sunlight exposition by also using dermatological creams with high protection and, frequently, by low therapeutic compliance. Vitamin D deficiency can have an important role in post-RTx's secondary HPT insurgence (25, 26). Also, in this case, immunosuppressive therapy has a role. GCs induce enzymes related to vitamin D catabolism and increase PTH and FGF23 levels (27). Also, CNIs appear to be inversely associated with vitamin D levels, maybe through inhibition of the 25-OH-ase activity of CYP3A4 by these drugs and downregulation of vitamin-D receptor (VDR) (28, 29).

Recently, it has been reported that low levels of 25-OH-VitD might be associated with worse graft outcomes, thus, promoting rejection episodes, possibly through the immunomodulatory mechanism exerted on the immune system (30). In any case, the direct and independent effect of 25-OH-VitD on graft outcome is still a matter of debate (26, 31–33).

#### Bone Disease

The most prevalent type of bone alteration in RTx-p is still debated. In this context, the bone biopsy might be considered the gold standard to obtain a precise bone evaluation in these patients. Unfortunately, at the moment, the indications and assessments of bone biopsy are difficult in clinical practise, and bone biopsy is performed only for research purposes. In a cohort of 20 RTx-p, adynamic bone disease (ABD) was the main lesion seen on biopsy specimen (34), while in a different group of 57 RTx-p, the main finding was osteitis fibrosa (35). This evident difference between these two studies may reflect the time in which the bone biopsy was performed, suggesting that RTx bone disease is a long-term evolving process. Recently, two other studies showed the same conflicting results. Neves et al. evaluated histomorphometric parameters in their cohort 2 years after RTx and the results revealed a mineralization defect and a cellular pattern consistent with HPT, with higher osteoid, osteoblastic, osteoclastic, and resorption surface. This pattern was, in part, attributable to hypovitaminosis D (5). On the contrary, Satu et al. conducted a study on 27 RTx-p, performing a bone biopsy at baseline and 2 years after RTx. They found in their cohort a marked decrease in bone turnover and an increase in abnormal bone mineralization. Moreover, they observed a significant decline in osteoblasts and osteoclasts activity and, thus, of bone formation rate. This happened in parallel with a sharp rise in Ca, 25-OH-VitD, and 1-25-OH-VitD, and a decrease in P, PTH, and osteocalcin levels. The abrupt lowering of serum PTH, combined with the fall in serum P levels, could be responsible for these changes in bone metabolism, although, the reason why higher vitamin D levels don't prevent abnormal mineralization remains unexplained (36).

In a very recent study by Jorgensen et al., performed in 97 RTx-p and followed up for 12 months after RTx, interesting results on this matter were shown. The histomorphometric study performed in this cohort showed an overall decrease in skeletal remodelling and bone turnover after RTx. Nevertheless, during the first year of RTx disorders of bone formation linked to HPT, such as excessive bone resorption and marrow fibrosis, were markedly decreased on histologic samples (37). Furthermore, in patients for whom it was possible to obtain dynamic histomorphometric data, similar findings were obtained with no significant changes in bone volume. This follows some evidence in literature where the bone undergoes an initial slowing in turnover rate, which lays the foundations for the subsequent increment in bone mass, mineralization, and overall health (1, 38). Cumulative steroid dosage was directly correlated with slower bone turnover and mineralization. Even if the wider use of steroid minimisation protocols has reduced the impact of GC on bone quality and density after-RTx, it appears that the negative effect of GC is exerted even with low exposure, with the most evident consequences on hip-MBD (37). Bone mineralization showed to be greatly influenced also by phosphate levels. The severity and the duration of hypophosphatemia negatively affected bone mineralization (37, 39). Since several factors that influence bone health, in a positive and a negative manner, come into play in the post-RTx period, it is readably understandable why there has been such great variability in the findings regarding post-RTx BMD.

Another important result highlighted in this study is the reliability of the dual-energy x-ray absorptiometry (DEXA) in evaluating bone mineralization. In this cohort of patients, DEXA findings were suggestive for substantial stability of MBD, especially in the axial skeleton. The main changes, found in a minority of the cohort, are a decrease in MBD at the distal radius and an increase in bone mass at the hip (37). These findings are in accordance with histomorphometric and biochemical findings, thus, corroborating the diagnostic and prognostic value of DEXA in the follow-up of RTx-BMD. Moreover, recent studies have highlighted that DEXA is reliable not only in assessing MBD but also in predicting patient's fracture risk, thus, giving the clinician a rapid and exhaustive overview of the bone status (40, 41).

Moreover, one of the most important findings of this study was the observation that some bone turnover markers (not subjected to significative renal clearance) were reliable markers in predicting the changes in bone histomorphometry. The most important markers are bone alkaline phosphatasis (BALP) (which reflects osteoblast activity), TRAP5b (which reflects osteoclasts number), and PINP (a fragment of pro-collagen laid down during bone deposition) (37, 42). These findings are of major importance because there is an urgent need for a non-invasive method to precisely assess bone status and activity. There is already some literature regarding these novel biomarkers, but more dedicated trials are needed to clarify whether these molecules could effectively be used as a “liquid biopsy” in the near future (43).

Regarding osteoporosis, the use of GCs remains the key risk factor for the development of this type of bone disease. An early post-RTx time is characterised by a rapid loss of bone mass that mainly affects trabecular bone due to intensive GC regimens (44). The GCs inhibit osteoblast proliferation and differentiation and stimulate apoptosis of both osteoblasts and osteoclasts. Moreover, they have indirect effects on the skeleton, inhibiting the synthesis of testosterone, oestrogen, and adrenal androgens. Lowering steroids exposure can reduce bone loss and should be especially considered for young patients, and, when possible, in RTx-p with severe and documented pre-RTx bone disease (45). Various studies have demonstrated that both early and late GC withdrawal can improve patients' bone health (46). Lastly, GCs have shown, *in vitro*, their capacity to enhance bone response to PTH (47). As underlined in two consecutive studies by Evenepoel et al. and Jorgensen et al., the GC cumulative dosage reduction of the latest immunosuppressive protocols had a great beneficial impact on bone health, favouring bone mineralization and reducing bone loss. Steroid minimisation protocols were associated with stable MBD after RTx, with significantly present bone loss only in the distal radius area (37, 38, 48). However, it must be remembered that even low doses of GC hamper bone metabolism, reducing bone formation and mineralization, and independently increasing the fracture risk (34, 49). Therefore, it is of foremost importance to aim for GC minimisation strategy, whenever possible, because of the great impact that this particular therapeutic approach could have on different aspects of patients' quality of life. A strong debate is present concerning the groups of patients, in which an early GC withdrawal is recommended. In our Unit in Milan, an accurate discussion for any case is made; the GS withdrawal is usually prescribed in presence of young recipients, as well as in patients with basal nephropathy with a low risk of recurrence (f.i. ADPKD) and, if possible, in patients with strong pre-RTx diabetes.

#### Vascular Calcifications

As previously described, the development of vascular calcifications (VC) is a dynamic and actively regulated process. By the time a patient requires RTx, endothelial dysfunction and atherosclerosis are common, with 50–60% of patients having detectable vascular calcifications (50). Despite RTx and amelioration of kidney function, calcification of the thoracic aorta and coronary arteries progresses around 4 and 11% per year, respectively (51). Increasing evidence is emerging regarding the influence of bone health and metabolism and vascular calcifications even in RTx-p. In a work performed by our group, the presence of aortic calcification was found in more than 60% of cases, and osteoprotegerin levels were inversely associated with VC prevalence and progression 12 months after RTx. A recent study by Sotomayor et al. performed in 678 RTx-p found that the prevalence of VC was 9% in patients with normal BMD, 11% in patients with osteopenia, and 25% in patients with osteoporosis. Moreover, higher BMD was associated with a lower risk of VC, independent of age, gender, body mass index, eGFR, and immunosuppressive therapy (52).

At present, several studies have reported an association between VC and mortality (53, 54). In a study by Lewis et al. in patients with RTx and kidney-pancreas transplantation, a significant association between VC and mortality was found. However, no correlation was reported with graft outcome (55). In another study performed by our group, a significant progression of coronary calcifications in a 5-year follow-up has been demonstrated. Both the presence of coronary calcifications and the progression were associated with cardiac events and death (56).

## Management of the Post-Transplant Bone Mineral Disorder

Despite the approach to post-RTx MBD being postulated on KDIGO guidelines, levels of recommendations are still not satisfying enough (45, 57). Thus, there is a need for stronger evidence to make it possible for researchers to delineate firmer guidelines for clinical practise. The following figure summarised the main principles for MBD treatment during RTx (Figure 2). In the following part, the review will focus on the pharmacologic agents of major interest in clinical practise. To help the reader, Tables 2, 3 were reports of the key readings about MBD post-RTx and the main therapeutic agents that have been reported.

**Table 2:** Key readings concerning post-RTx-MBD.

**KEY READINGS**	
**MBD topic**	**Main readings**
Bone histomorphometry	-Keronen S, Martola L, Finne P, Burton IS, Kröger H, Honkanen E. Changes in Bone Histomorphometry after Kidney Transplantation. Clin J Am Soc Nephrol. 2019 Jun 7;14(6):894–903.
Vitamin D	-Alfieri C, Ruzhytska O, Vettoretti S, Caldiroli L, Cozzolino M, Messa P. Native Hypovitaminosis D in CKD Patients: From Experimental Evidence to Clinical Practise. Nutrients. 2019 Aug 15;11(8):1918 -Stavroulopoulos A, Cassidy MJ, Porter CJ, Hosking DJ, Roe SD. Vitamin D status in renal transplant recipients. Am J Transplant. 2007 Nov;7(11):2546–52. -McGregor R, Li G, Penny H, Lombardi G, Afzali B, Goldsmith DJ. Vitamin D in renal transplantation - from biological mechanisms to clinical benefits. Am J Transplant. 2014 Jun;14(6):1259–70.
Calcimimetics	-Alfieri C, Mattinzoli D, Messa P. Tertiary and Postrenal Transplantation Hyperparathyroidism. Endocrinol Metab Clin North Am. 2021 Dec;50(4):649–662. -Evenepoel P, Cooper K, Holdaas H, Messa P, et al. A randomised study evaluating cinacalcet to treat hypercalcemia in renal transplant recipients with persistent hyperparathyroidism. Am J Transplant. 2014 Nov;14(11):2545–55. -Courbebaisse M, Diet C, Timsit MO, Mamzer MF, Thervet E, Noel LH, Legendre C, Friedlander G, Martinez F, Prié D. Effects of cinacalcet in renal transplant patients with hyperparathyroidism. Am J Nephrol. 2012;35(4):341–8.
Bisphosphonates	-Coco M, Glicklich D, Faugere MC, Burris L, Bognar I, Durkin P, Tellis V, Greenstein S, Schechner R, Figueroa K, McDonough P, Wang G, Malluche H. Prevention of bone loss in renal transplant recipients: a prospective, randomised trial of intravenous pamidronate. J Am Soc Nephrol. 2003 Oct;14(10):2669–76 -Smerud KT, Dolgos S, Olsen IC, Åsberg A, Sagedal S, Reisæter AV, Midtvedt K, Pfeffer P, Ueland T, Godang K, Bollerslev J, Hartmann A. A 1-year randomised, double-blind, placebo-controlled study of intravenous ibandronate on bone loss following renal transplantation. Am J Transplant. 2012 Dec;12(12):3316–25
Denosumab	-Alfieri C, Binda V, Malvica S, et al. Bone Effect and Safety of One-Year Denosumab Therapy in a Cohort of Renal Transplanted Patients: An Observational Monocentric Study. J Clin Med. 2021 May 6;10(9):1989. -Bonani M, Frey D, Brockmann J, Fehr T, Mueller TF, Saleh L, von Eckardstein A, Graf N, Wüthrich RP. Effect of Twice-Yearly Denosumab on Prevention of Bone Mineral Density Loss in De Novo Kidney Transplant Recipients: A Randomised Controlled Trial. Am J Transplant. 2016 Jun;16(6):1882–91

*MBD, mineral bone disorder*.

**Table 3:** Pharmacologic characteristics of the main therapeutical agents for RTx-MBD.

**Pharmacologic Agent**	**Therapeutic indications**	**Adverse effects**
Vitamin D	Surgical hypoparathyroidism; Osteoporosis, prevention; Vitamin D insufficiency/deficiency;	hypercalcemia, hypercalcuria, hyperphosphatemia, excessively low PTH, calcification of soft tissues
Calcimimetics	Hyperparathyroidism, primary Hyperparathyroidism, tertiary Parathyroid carcinoma	ABD, Hyocalcemia related effects: QT prolongation and ventricular arrhythmia; seizure disorder Caution in moderate-to-severe hepatic impairment;
Bisphosphonates	High bone turnover states (osteoporosis, HPT, malignancies, bone metastasis)	Osteonecrosis, ABD, hypocalcemia, long half-life (10 years), not safe if eGFR <30 ml/min
Denosumab	High bone turnover states (osteoporosis, HPT, malignancies, bone metastasis)	Osteonecrosis, ABD, urinary infections
Teriparatide	Osteoporosis in postmenopausal females with high risk for fracture. Treatment to increase bone mass in males with primary or hypogonadal osteoporosis with high risk for fracture. Treatment of glucocorticoid-induced osteoporosis with high risk for fracture	Hypercalcemia Nausea Worsening of cutaneous calcification or calciphylaxis Orthostatic hypotension Avoid use in patients with Paget disease, bone metastases or a history of skeletal malignancies

*PTH, parathormone; ABD, adynamic bone disease; eGFR, estimated glomerular filtration rate*.

### Vitamin D Supplementation

Vitamin D supplementation can lower bone loss both in the femoral and lumbar regions, but nowadays, evidence regarding the eventual effect on fracture rate is still lacking. Moreover, vitamin D has well-known pleiotropic effects on the renin-angiotensin system and immune-modulatory power on T and dendritic cells, virtually influencing graft function and outcome (58). Data on the effect of vitamin D supplementation on post-RTx MBD are controversial. Recent evidence has found that the administration of 25,000 UI of cholecalciferol was effective in reducing PTH levels, but not in ameliorating MBD (59, 60). However, in other studies, when vitamin D was administered in combination with calcium supplementation, preservation of BMD in various sites was observed. In particular, one report indicated that vitamin D 400 UI/die, combined with 600 mg/die of oral calcium, was associated at 12 months with a reduction in lumbar spine, femoral neck, and total hip bone loss (61). A possible dosage of 25-OH-VitD might be 400 UI/die in RTx-p with CKD G1-3T (62). Nevertheless, there has been little research investigating the impact of Vitamin D levels and supplementation on fractures incidence. A recent retrospective observational trial found out that higher supplementation of Vitamin D levels was associated with a reduction in vitamin deficiency and a pronounced reduction in fracture rate, from 9.1 to 3.1% (63). All these studies suggest that supplementation of vitamin D, both active and inactive forms could ameliorate post-RTx MBD, but more research on this topic is needed to better delineate the effects of vitamin D on bone homeostasis in RTx-p.

Paracalcitol has shown similar effects to native vitamin D but was linked to a higher risk of hypercalcemia (64). A randomised study found a protective effect of paricalcitol on bone remodelling, with a reduction in bone alkaline phosphatasis (bALP) and osteocalcin in association with improvements in lumbar and spine BMD (65).

### Calcimimetics

Cinacalcet is a positive allosteric modulator of calcium-sensing receptor (CaSr). In a study concerning Hyper-Ca and secondary HPT, performed on 114 RTx-p, cinacalcet effectively reduced PTH and calcium and determined an increase in serum phosphorus without any adverse effects on graft function compared to placebo (66). Recently, Bernador et al. performed a multicentre study that involved 20 paediatric RTx-p with SHPT. This work showed a significant and linear relationship between cinacalcet dose and therapy duration, PTH and calcium reduction, and phosphorus increase. Most importantly, no significant impact on eGFR was found (67). A recent study performed by Hyang et al., performed on 9 RTx-p with tertiary HPT showed conflicting results. Cinacalcet was able to normalise serum calcium and phosphorus levels but failed to normalise PTH in those patients who had very high baseline PTH levels (68). This may imply that more studies are needed to ascertain if cinacalcet could be a definitive and effective alternative to parathyroidectomy in RTx-p. Moreover, in other studies, it has been observed that cinacalcet had no positive effects on BMD even if it suppresses PTH. This might be due to the concomitant sensitisation of calcium-sensing receptors in the bone, which may counteract benefits deriving from lower PTH levels (69). An important side effect of cinacalcet is that of inducing hypercalciuria and consequent nephrocalcinosis, even if a small study involving 34 RTx-p found no lesions of this kind in biopsies conducted at 3 and 12 months after RTx (70). Moreover, the effect of cinacalcet on bone histomorphometry has not been extensively studied yet.

### Antiresorptive Agents

Bisphosphonates and denosumab are common agents widely used in osteoporosis. They have to be prescribed carefully in RTx-p, especially in patients who are at risk of ABD, and, thus, a bone biopsy could be considered before the start of these agents (45).

Bisphosphonates bind strongly to the mineralized bone, inhibiting osteoclasts' action and inducing apoptosis of these cells. Their half-life is particularly long, about 10 years. About 50% of the drug is not taken up by the bone and is cleared by the kidney *via* glomerular filtration and tubular secretion. Thus, the safety of these drugs strongly depends on kidney function. For this reason, they are considered safe if used in presence of an eGFR > 30 ml/min (45).

Most of the studies conducted on bisphosphonates in RTx-p showed preservation of lumbar spine and femoral neck BMD in the early post-RTx period. Furthermore, ABD has not been a widely observed phenomenon. In a study conducted by Coco et al., pamidronate, combined with calcium and calcitriol supplementation, led to the preservation of vertebral BMD, but increased the low turnover rate on bone biopsy (71). A a few years later the same group studied the effects of risedronate on BMD. They found that this drug was not associated with an increased risk of ABD but did not affect the preservation of BMD either (56). This may reflect different drug power, with second-generation bisphosphonates (pamidronate), having a stronger enzymatic inhibition ability than the third generation (risendronate).

A series of studies, in which vitamin D was part of the standard care or where there was a reduced GCs load, failed to show bisphosphonates' benefits on BMD (72). This suggests that nowadays, considering the widespread use of vitamin D and low GCs exposure, bisphosphonates could be reserved only for patients who are at high-risk.

Denosumab is a humanised monoclonal antibody directed against the receptor activator of the Nf-kB ligand. It reduces bone resorption, significantly increases BMD, and decreases the risk of fracture in women with osteoporosis (73).

Its efficacy has also been proven in patients with severe impairment of kidney function (74). Different to bisphosphonates, Denosumab is not cleared, so it is an attractive therapy in CKD and RTx-p. Recently, interesting studies have emerged about its use in RTx-p. Bonani et al. performed a randomised case-control trial on the denosumab effect on a cohort of 90 RTx-p. They administered the drug twice a year for the first two years of RTx. They found that treatment with denosumab was associated with increased BMD at 12 months in both lumbar spine and hip; while at 6 months, only the lumbar spine showed significant benefits on BMD. Moreover, bone turnover decreased significantly in the denosumab group, whereas it remained unchanged in the control group. Infections were significantly more frequent in the denosumab group, and they were mainly of bacterial aetiology, with UTI being the most frequent. Viral infections showed no difference between the two groups. Hypocalcaemia was more frequent in the denosumab group (75). A study performed by our group on 32 RTx-p showed similar results. After 12 months of denosumab therapy, there was a significant improvement of T score both at vertebral and femoral neck sites. The improvement was so significant that caused a reduction in the number of patients affected by osteoporosis, because many patients shifted to osteopenia class, according to T-score. No significant relationship between denosumab and hypocalcaemia and UTI was found in our cohort (76). A recent meta-analysis confirmed the results observed in these studies. Denosumab was associated with increased BMD at 12 months at the lumbar spine and femoral neck. Hypocalcaemia was a frequent adverse effect, but all reported episodes were mild and asymptomatic, requiring only Ca and vitamin D supplementation. Infections were confirmed to be another frequent complication following denosumab administration; in particular, it was confirmed an increased frequency of diarrhoea and UTI. However, no differences in RTx function and rejection were found (77).

### Teriparatide

This anabolic agent can improve BMD in RTx-p with GC-induced osteoporosis. However, in a study conducted on 26 RTx-p with a FU time of 6 months, patients who received teriparatide did not show improvement of bone density in the lumbar spine and distal radius; still, there was a stabilisation of BMD in the femoral neck (78). Although teriparatide could be useful for patients with osteoporosis and ABD, given the cost of the drug, more RCTs are needed to delineate solid indications for its use in RTx-p.

## Conclusions

The RTx is characterised by several mineral and bone anomalies, resulting in loss of bone density, increased risk of fracture, and potentially increased risk of mortality. The correct treatment of this condition is strictly determined by a deep and careful evaluation of biochemical anomalies and of the pre- and post-RTx MBD-related factors (f.i. immunosuppression, anticoagulant therapy, etc.) present in any specific case. The disorders might be sometimes difficult to be treated, but fortunately, at the moment, many different therapeutic options are available. In the future, to obtain an ever-greater individualisation of therapy, an increase of the evidence and the specificity of international guidelines and more uniform management of these anomalies worldwide should be expected.

## Author Contributions

PMo, CA, DM, MC, AC, EF, SM, PMe, and GC actively contributed to the manuscript by writing individual sections of the final article. All authors contributed to the article and approved the submitted version.

## Conflict of Interest

The authors declare that the research was conducted in the absence of any commercial or financial relationships that could be construed as a potential conflict of interest.

## Publisher's Note

All claims expressed in this article are solely those of the authors and do not necessarily represent those of their affiliated organizations, or those of the publisher, the editors and the reviewers. Any product that may be evaluated in this article, or claim that may be made by its manufacturer, is not guaranteed or endorsed by the publisher.
